# The Carboxyl-Terminus of Human Immunodeficiency Virus Type 2 Circulating Recombinant form 01_AB Capsid Protein Affects Sensitivity to Human TRIM5α

**DOI:** 10.1371/journal.pone.0047757

**Published:** 2012-10-19

**Authors:** Tadashi Miyamoto, Emi E. Nakayama, Masaru Yokoyama, Shiro Ibe, Shunpei Takehara, Ken Kono, Yoshiyuki Yokomaku, Massimo Pizzato, Jeremy Luban, Wataru Sugiura, Hironori Sato, Tatsuo Shioda

**Affiliations:** 1 Department of Viral Infections, Research Institute for Microbial Diseases, Osaka University, Suita Osaka, Japan; 2 Laboratory of Viral Genomics, Pathogen Genomics Center, National Institute of Infectious Diseases, Musashimurayama, Tokyo, Japan; 3 Department of Infection and Immunology, Clinical Research Center, National Hospital Organization Nagoya Medical Center, Naka-ku, Nagoya, Aichi, Japan; 4 Department of Microbiology and Molecular Medicine, University of Geneva, Geneva, Switzerland; Dana-Farber Cancer Institute, United States of America

## Abstract

Human immunodeficiency virus (HIV) type 2 shows limited geographical distribution compared with HIV type 1. Although 8 genetic groups of HIV type 2 (HIV-2) have been described, recombinant viruses between these groups are rarely observed. Recently, three HIV-2 patients in Japan were described with rapidly progressive, acquired immunodeficiency. These patients were infected with an A/B inter-group recombinant designated CRF01_AB. Here, we characterize the capsid protein (CA) encoded by the viruses from these patients. HIV-2 CRF01_AB CA showed unique amino acid sequence almost equally distinct from group A and group B viruses. Notably, HIV-2 CRF01_AB CA showed potent resistance to human TRIM5α. In addition to the previously identified amino acid position 119 in the N-terminal domain of CA, we found that HIV-2 CRF01_AB-specific amino acid substitutions in the C-terminal domain also were necessary for resistance to human TRIM5α. These results indicate that retroviruses can evade TRIM5α by substitution at residues within the C-terminal domain of CA.

## Introduction

Human immunodeficiency virus type 2 (HIV-2) has been detected primarily in West Africa, in contrast to the global distribution of the type 1 epidemic virus (HIV-1). Based on molecular evidence, HIV-2 and HIV-1 are presumed to derive from simian immunodeficiency viruses that originated in sooty mangabey (SIVsm) and chimpanzee (SIVcpz), respectively, as a result of zoonotic transfer between non-human primates and human. The HIV-1 and HIV-2 bear a considerable degree of homology in both gene organization and RNA sequence (30%–60%) [1]–[4]. It is generally believed that HIV-2 is less pathogenic than HIV-1. However, certain HIV-2 patients with high plasma HIV-2 loads develop acquired immune deficiency syndrome (AIDS) as rapidly as HIV-1 patients do [4]. To date, eight HIV-2 groups have been distinguished on the basis of phylogenetic (sequence) analysis; each group is presumed to have originated from an independent zoonotic event [5].

TRIM5α was identified as a factor that restricts HIV-1 infection in rhesus monkey (Rh) cells [6]. TRIM5α is thought to degrade the core of the incoming virus [7], [8]. TRIM5 proteins are members of the tripartite motif family containing RING, B-box, and coiled-coil domains. The alpha isoform of TRIM5 has an additional C-terminal PRYSPRY (B30.2) domain [9]. In cynomolgus monkey (CM), TRIM5α also has been demonstrated to restrict HIV-1 infection [6], [10]. In contrast, the human TRIM5α exhibits minimal restriction of HIV-1 infection [11]–[14], but shows moderate levels of restriction for HIV-2 [15].

Capsid (CA) proteins are components of the viral core; the CAs of HIV-1 and HIV-2 have similar primary and three dimensional structures [16]. CA is composed of a surface-exposed N-terminal domain (NTD) and a C-terminal domain (CTD) that is required for oligomerization [17]. We previously identified a single amino acid of the HIV-2 capsid that determines the susceptibility of HIV-2 to CM TRIM5α. Viruses that encoded CAs with either alanine or glutamine at amino acid residue 119 (which corresponded to the 120th amino acid of the CA of the GH123 viral strain) could grow in cells harboring the CM TRIM5α. In contrast, HIV-2 encoding CA with proline at the same position showed restricted growth in cells harboring the CM TRIM5α. Similar results, although to a lesser extent, were observed when the human TRIM5α was used [15]. Furthermore, an analysis of HIV-2 CA variation in a West African Caio cohort demonstrated that the presence of proline at CA positions 119, 159, and 178 was more frequent in individuals with lower viral loads (VLs); the presence of non-proline residues at all 3 residues was more frequent in individuals with high VLs. The *in vitro* replication levels of viruses bearing changes at the 3 positions suggested that these 3 residues influence virus replication by altering susceptibility to TRIM5α [18]. These results also suggested that TRIM5α controls virus replication in HIV-2-infected individuals.

Recently, five HIV-2-seropositive cases were identified in Japan. Three isolates (NMC307, NMC716, and NMC842) were recovered from these patients and were shown by full-length genomic analysis to represent a recombinant (designated HIV-2 CRF01_AB) of group A and B strains [19]. Although more than 75% of patients with HIV-2 have asymptomatic prognoses throughout their lifetimes [1], [20], all 3 of the CRF01_AB patients were found to be at an advanced stage of AIDS with low CD4+ cell counts and high HIV-2 VLs [19]. All 3 patients were under 40 years of age when first diagnosed as HIV-2 positive [19]. Assessment of risk factors suggested that all three were infected via heterosexual contacts; no personal connection was confirmed among any of these cases [19]. In the present study, we characterized the HIV-2 CRF01_AB CA obtained from these patients and found several unique properties of HIV-2 CRF01_AB, including potent resistance to human TRIM5α-mediated restriction.

## Results

### HIV-2 CRF01_AB Strains Show Unique CA Sequences

**Figure 1:**
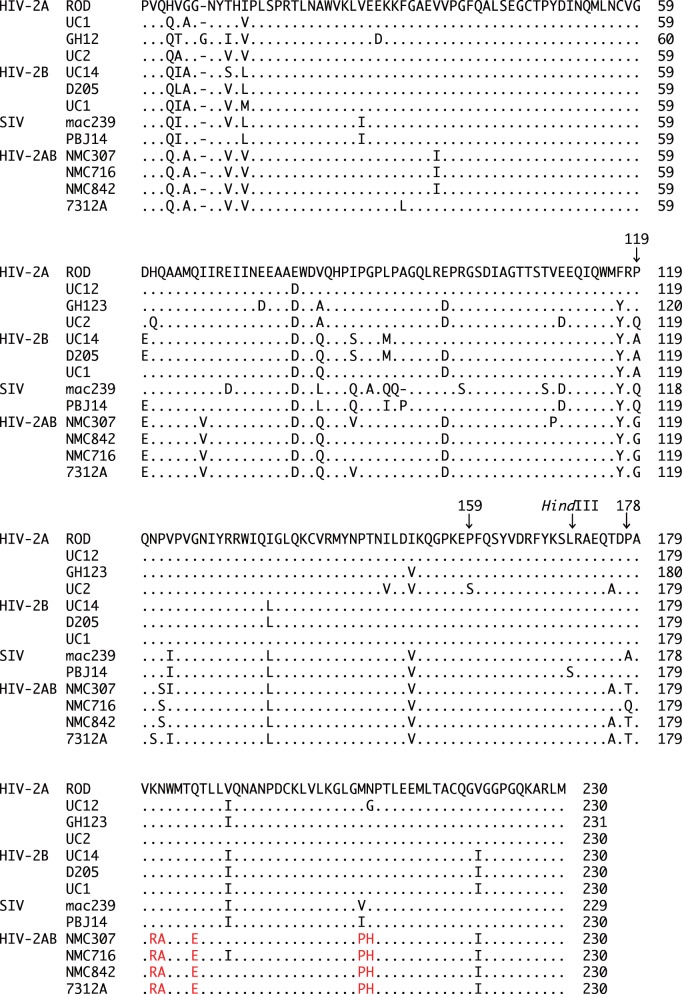
Alignments of amino acid sequences of CA proteins encoded by selected HIV-2 isolates and SIV from the Los Alamos databases. Dots denote amino acid identity with the ROD CA; dashes denote gaps introduced to optimize alignment. HIV-2 CRF01_AB-specific amino acid residues are in red. Arrows indicate key residues at 119, 159, and 178, and the position (in the corresponding DNA sequence) of the *Hind*III restriction site used in the constructs. HIV-2A, HIV-2B, and HIV-2AB denote HIV-2 group A, HIV-2 group B, and HIV-2 CRF01_AB, respectively.

**Figure 2:**
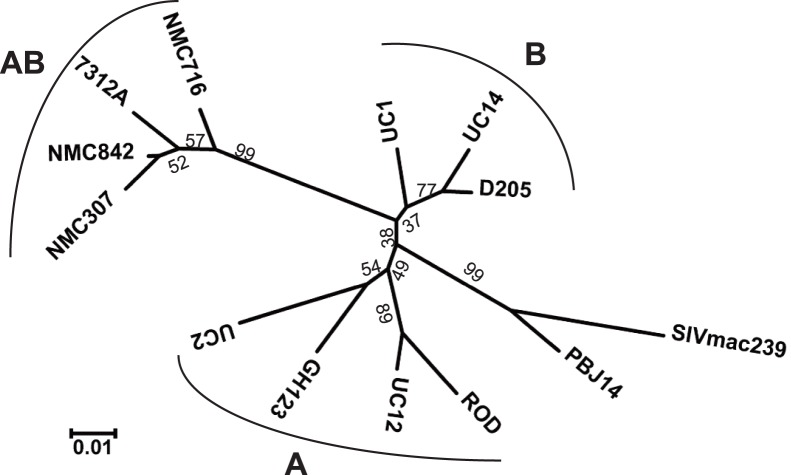
Phylogenetic tree of HIV-2 isolates and SIV. This phylogenetic tree was constructed by the neighbor-joining method. Bootstrap probabilities (%), as calculated by 1000 iterations, are shown at the major tree nodes. Scale bar represents 0.01 amino acid substitutions per site. A, B, and AB denote HIV-2 group A, HIV-2 group B, and HIV-2 CRF01_AB, respectively.

Fig. 1 shows an alignment of the deduced amino acid sequences of the CAs of HIV-2 group A (ROD, UC12, GH123, and UC2), HIV-2 group B (UC14, D205, and UC1), SIVs (SIVmac239 and SIVsm PBJ14), and HIV-2 CRF01_AB (NMC307, NMC716, NMC842, and 7312A). As we reported previously [15], [18], the 119th amino acid position is a proline, glutamine, or alanine in the CAs of HIV-2 group A, HIV-2 group B, and SIVs. However, the CAs of the HIV-2 CRF01_AB strains uniquely possess a glycine at this position. Based on the genomic structure of HIV-2 CRF01_AB, A/B recombinant breakpoints within this isolate are located near or within the *env* gene, such that HIV-2 CRF01_AB can be considered to consist of a group B backbone that incorporates group A *env* fragments [19]. These presumed breakpoints could be taken to suggest that CRF01_AB CA should be encoded as a B-like sequence. However, phylogenetic analysis of these CA sequences (Fig. 2) reveals that the deduced HIV-2 CRF01_AB CA proteins constitute a distinct cluster, with the dendrogram exhibiting a long branch length compared to the CAs of HIV-2 group A, HIV-2 group B, and SIV.

### HIV-2 CRF01_AB CA is Highly Resistant to Human TRIM5α

**Figure 3:**
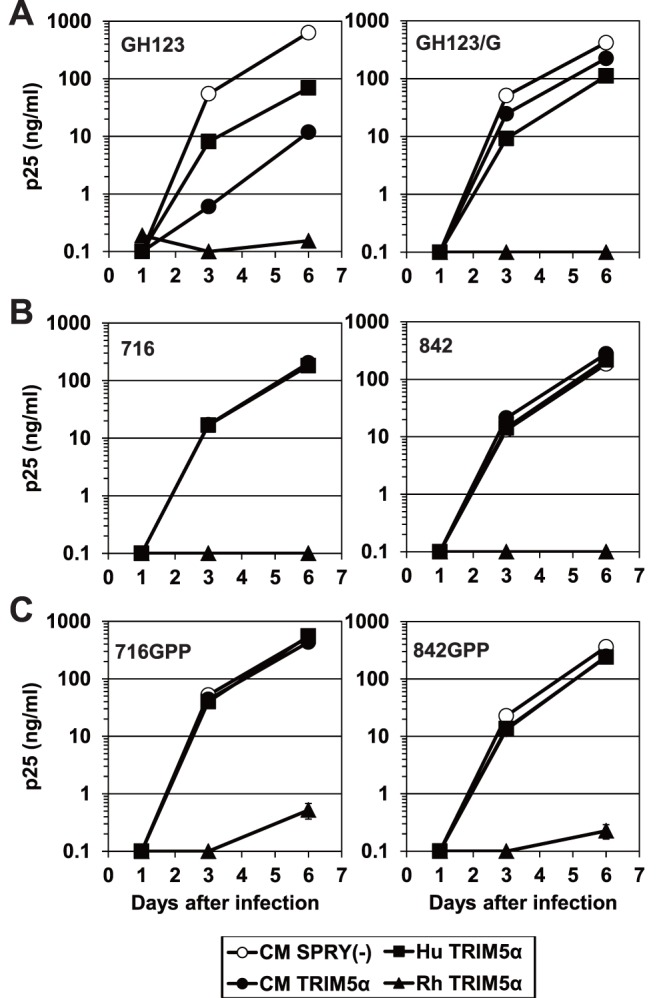
Growth of HIV-2 strain GH123 and variants thereof in the presence of TRIM5α. (A), (B), (C) Virus levels were measured by ELISA detection of p25 (CA) levels in supernatants. CEM-SS cells were infected with recombinant SeV encoding rhesus (Rh: black triangles); cynomolgus monkey (CM: black circles); human (Hu: black squares); or CM SPRY(-) (white circles) TRIM5α. CM SPRY (-) has a dominant negative effect on the anti-viral activity of TRIM5α and serves as a negative control. Nine hours after infection, cells were superinfected with GH123, GH123/G, 716, 842, 716GPP, or 842GPP. Error bars show actual fluctuations between levels of p25 (CA) in duplicate samples from one of three independent experiments.

In a previous study, we reported that the amino acid at residue 119 of the HIV-2 CA affects susceptibility to the restriction of virus replication by CM and human TRIM5α [15], such that HIV-2 encoding CA(Pro119) was sensitive to CM and human TRIM5α, while HIV-2 encoding CA(Gln119) or CA(Ala119) was resistant [15]. We also reported that mutation of HIV-2 strain GH123 to encode glycine at the corresponding position (GH123/G) rendered GH123 resistant to CM TRIM5α [21]. To further test the role of the CA protein in TRIM5α resistance, we generated recombinant versions of the GH123 virus (716 or 842) in which the CA-encoding segment of *gag* was replaced with that of the A/B recombinants NMC716 or NMC842 (respectively). We used a recombinant Sendai virus (SeV) system to express CM, Rh, and human TRIM5α and CM TRIM5α lacking the PRYSPRY domain as a negative control (Fig. S1). In the presence of CM TRIM5α, infection by the parental GH123 virus was restricted, but infection by GH123/G was resistant to CM TRIM5α-mediated restriction (Fig. 3A). Infection by 716 or 842 was resistant to CM TRIM5α (Fig. 3B). In contrast, infection by any of the 4 variants (GH123, GH123/G, 716, and 842) was completely restricted by Rh TRIM5α (Fig. 3A, B). These results for cells producing CM or Rh TRIM5α are consistent with our previous findings [22]. In cells producing human TRIM5α, the replication of parental GH123 and of the GH123/G mutant were partially restricted (Fig. 3A), while 716 and 842 replicated as efficiently as in negative control cells that did not produce a functional TRIM5α (Fig. 3B). The mean ratios of the p25 levels at 6 days after infection in the cells producing human TRIM5α to those in the negative control cells were 0.14 for GH123, 0.30 for GH123/G, but 0.81 for 716 and 1.02 for 842 in three independent experiments. The ratio of GH123/G was significantly higher than that of GH123 (P = 0.0086, t test) but lower than those of 716 (P = 0.0059, t test) and 842 (P = 0.0030, t test). Similar results were obtained when we calculated the mean ratios of the p25 levels at 3 days after infection (data not shown). These data indicate that the CA sequences of the CRF01_AB strains conferred higher potential to escape from human TRIM5α than those of GH123/G.

### Viral Sensitivity to Human TRIM5α-mediated Restriction in a Single Round Infection Assay

**Figure 4:**
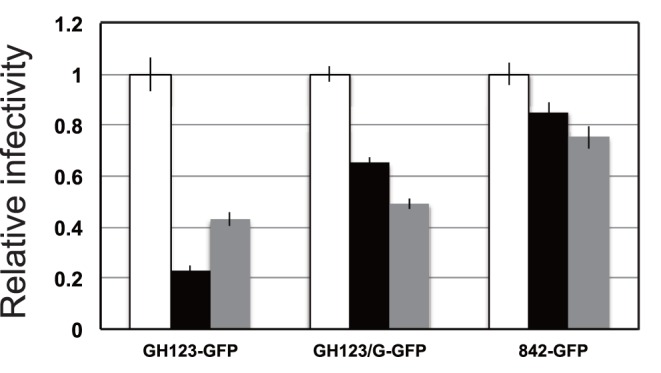
Viral sensitivity to TRIM5α-mediated restriction in a single-round infection assay. Feline CRFK cells were infected with SeV encoding cynomolgus monkey (CM; black bars), human (grey bars), or CM SPRY(-) (white bars) TRIM5α. CM SPRY (-) has a dominant negative effect on the anti-viral activity of TRIM5α and serves as a negative control. The cells then were superinfected with a green fluorescent protein (GFP)-expressing virus, GH123-GFP, GH123/G-GFP, or 842-GFP containing 500ng of p25 (CA). Two days after infection, the cells were fixed by formaldehyde, and GFP-producing cells were counted by flow cytometry. Numbers of GFP-positive cells in CM SPRY (-)-producing cells are set at one and relative numbers to CM SPRY (-) of GFP-positive cells are shown. Error bars denote standard deviations of triplicate samples from one of three independent experiments.

TRIM5α restricts viral infection at a post-entry step [6], [23], [24]. To focus on early steps of virus replication, we performed a single-round infection assay, in which infection is detected as fluorescence generated by production of the green fluorescent protein (GFP). To construct mutant viruses encoding GFP, the fragment of GH123, 842, or GH123/G that encoded the matrix (MA) and CA proteins was transferred to the *env*-disrupted HIV-2 genomic clone pROD-env(-)-GFP, which directs the production of GFP after infection [25]. Vesicular stomatitis virus glycoprotein (VSV-G) pseudotyped wild-type and mutant HIV-2 GFP viruses were inoculated into feline CRFK cells producing TRIM5α, and GFP-positive cells were counted 2 days after infection. In this experiment, we used feline cells, since feline cells lack expression of endogenous TRIM5α. In the presence of CM TRIM5α, the numbers of GFP-positive cells were greater in cells infected with GFP-expressing viruses encoding the GH123/G or 842 CAs than in those infected with the GFP-expressing viruses encoding GH123 CA (Fig. 4), confirming that viruses encoding CA(G119) were resistant to CM TRIM5α. Consistent with the results shown in Fig. 3B, the GFP-expressing virus encoding the 842 CA from a patient was more resistant to human TRIM5α-mediated restriction than viruses encoding the CAs from GH123 (P = 0.0010, t-test) or GH123/G (P = 0.0026, t-test) (Fig. 4).

### Viral Growth in TRIM5α Knock-down Cells

**Figure 5:**
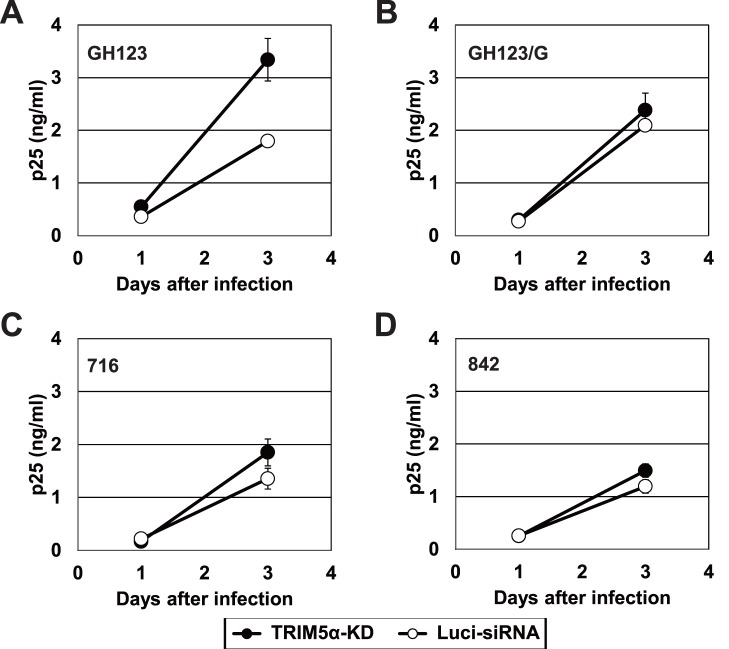
Viral growth in TRIM5α knock-down cells. (A), (B), (C) and (D) TRIM5α-KD Jurkat (“knock-down”) or Luci-siRNA Jurkat (control) cells were infected with derivatives of GH123 virus. Culture supernatants were periodically assayed for levels of virus capsid. Error bars show actual fluctuations of duplicate samples from one of two independent experiments. Black and white bars denote TRIM5α-KD Jurkat and Luci-siRNA Jurkat cells, respectively.

We next investigated whether the different resistance to human TRIM5α restriction among recombinant HIV-2 strains still applied in cells producing physiological levels of human TRIM5α protein. For this purpose, we used TRIM5α “knock-down” Jurkat cells (TRIM5α-KD Jurkat) and the corresponding control Jurkat line (Luci-siRNA Jurkat) [26]. It was demonstrated that the level of TRIM5α mRNA in TRIM5α-KD Jurkat is five times lower than that of Luci-siRNA Jurkat by TaqMan quantitative PCR. Three days after infection, GH123 replicated better in TRIM5α-KD Jurkat than in Luci-siRNA Jurkat (Fig. 5A). On the other hand, GH123/G, 716, and 842 yielded comparable titers in both cell lines (Fig. 5B, 5C, and 5D). In this experiment, we found that GH123/G also was resistant to human TRIM5α. Nevertheless, the data presented in Fig. 5 indicated that GH123 was sensitive to human TRIM5α produced at physiologically relevant levels, while 716 and 842 possessed potent resistance against human TRIM5α. Since TRIM5α-KD Jurkat always showed reduced proliferative properties compared to Luci-siRNA Jurkat (data not shown), presumably due to reduced TRIM5α levels [27], the p25 levels of all these viruses in Luci-siRNA Jurkat became higher than those in TRIM5α-KD Jurkat at 10 days after infection (data not shown).

### HIV-2 CRF01_AB CA C-terminal Domain-specific Sequence also Affects Viral Sensitivity to Human TRIM5α

We previously reported that the presence of proline at CA positions 119, 159, and 178 is more frequent in individuals with lower VLs [18]. Viral isolates NMC307, NMC716, and NMC842 all encoded CAs with proline at the 159th position (Fig. 1). However, the 178th amino acid residue was encoded as a threonine (NMC307 and NMC842) or as a glutamic acid (NMC716) in these isolates (Fig. 1). To test whether a single residue at amino acid 178 of HIV-2 CRF01_AB CA affects the sensitivity to human TRIM5α, we generated recombinant 716 or 842 viruses (designated 716GPP or 842GPP, respectively) that encoded CA (Pro178) proteins. As shown in Fig. 3C, 716GPP and 842GPP escaped from human TRIM5α restriction as efficiently as 716 and 842 did. These data suggest the existence of viral determinants for human TRIM5α-resistance other than the previously identified 119th and 178th amino acid positions of CA.

**Figure 6:**
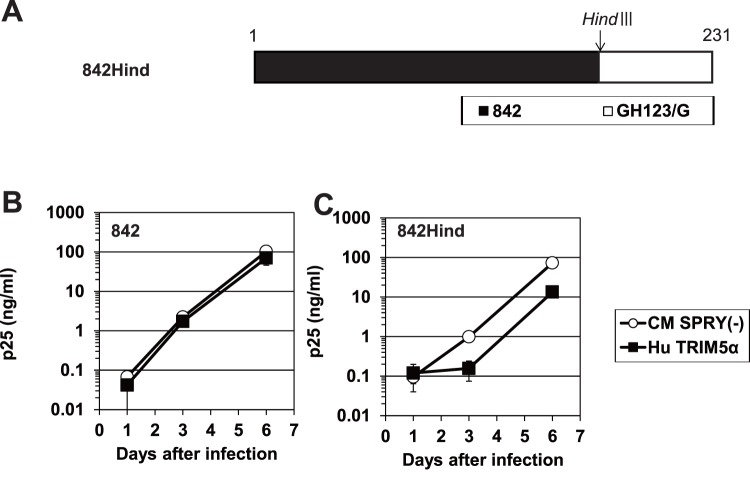
HIV-2 CRF01_AB CA C-terminal domain-specific sequence also affects viral sensitivity to human TRIM5α. (A) Schematic representation of chimeric viral CAs. Black and white bars show 842 and GH123/G CA peptide sequences, respectively. An arrow denotes the position (in the corresponding DNA sequence) of the *Hind*III restriction site used in the construct. (B and C) CEM-SS cells were infected with recombinant SeV encoding human (Hu: black squares) or CM SPRY(-) (white circles) TRIM5α. Nine hours after infection, cells were superinfected with 842 (B) and 842Hind (C). Culture supernatants were assayed for levels of p25 (CA). Error bars show actual fluctuations between levels of p25 (CA) in duplicate samples from one of three independent experiments.

To search for the viral determinants of human TRIM5α resistance other than the 119th and 178th amino acid positions of HIV-2 CA, we constructed a chimeric virus 842Hind by replacing the segment of the 842 genome that encodes CA C-terminal residues 170 to 231 with the corresponding region of GH123 (Fig. 6A). When tested in cells that produced human TRIM5α, 842 was strongly resistant to human TRIM5α as expected (Fig. 6B). However, the 842Hind construct, which encoded the NMC842 CA with the GH123 CA C-terminal short region, lost this resistance to human TRIM5α (Fig. 6C). The mean ratios of the p25 levels at 6 days after infection in the cells producing human TRIM5α to those in the negative control cells were 0.73 for 842 and 0.16 for 842Hind in three independent experiments. The ratio of 842Hind was significantly lower than that of 842 (P = 0.0003, t test). Similar results were obtained when we calculated the mean ratios of the p25 levels at 3 days after infection (data not shown). These results suggest that one or more of the HIV-2 CRF01_AB-specific amino acid residues within the CA C-terminal short region (Fig. 1, shown in red) also are necessary to fully evade human TRIM5α.

### Molecular Dynamics of N-terminal Domain (NTD) of HIV-2 CRF01_AB CA

**Figure 7:**
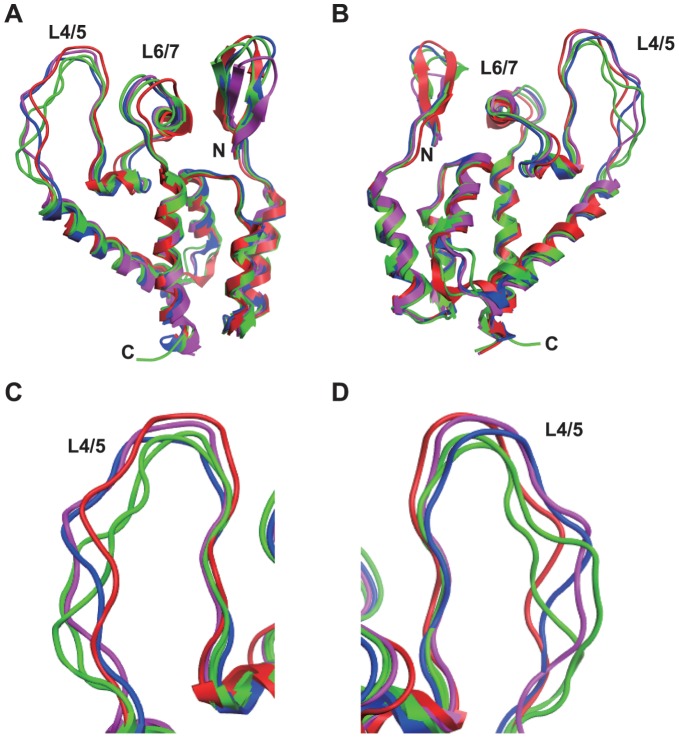
Structural models of the HIV-2 capsid N-terminal domain. Models were constructed by homology modeling and molecular dynamics simulations with the high-resolution X-ray crystal structure of the HIV-2 capsid N-terminal domain (CA NTD) (PDB code: 2WLV [16]) as the starting structure. Averaged conformations of the overall structure of the CA NTD (from the amino acid position 1 to 150) during 5–20 nanoseconds of MD simulations (A and B) and a close-up view around the L4/5 loop (C and D) are indicated. N and C indicate the amino termini and carboxyl termini, respectively. Models are color coded as follows: red, 842; blue, GH123/G; green, CM TRIM5α-resistant viruses (GH123/Q and GH123/A); and purple, CM TRIM5α-sensitive virus (GH123/P).

Residue 120 of the GH123 CA, which corresponds to residue 119 of the CRF01_AB CA, is located in the loop between α-helices 6 and 7 (L6/7) of CA NTD. Our previous molecular dynamics simulation study of HIV-2 CA NTD revealed that mutations at this position affected conformation of the neighboring loop between α-helices 4 and 5 (L4/5), and TRIM5α-sensitive viruses were predicted to share a common L4/5 conformation. In addition, the shared L4/5 structures of the sensitive viruses were associated with a decreased probability of hydrogen bond formation between GH123 CA’s Asp97 (in L4/5) and Arg119 (corresponding to residue 118 in HIV-2 CRF01_AB CA; in L6/7) [21]. TRIM5α-resistant viruses exhibited a variable L4/5 conformation and a higher probability of hydrogen bond formation between L4/5 and L6/7 [21]. As noted above, HIV-2 CRF01_AB strains have a unique Gly119 (Fig. 1), which we had not previously modeled by molecular dynamics simulation. Therefore, three-dimensional (3-D) models of HIV-2 GH123/G and NMC842 CA NTD were constructed using homology modeling based on the crystal structures of the HIV-2 CA NTD, and the models were subjected to molecular dynamics simulation to compare the results with those derived from previously constructed 3-D structural models of TRIM5α-sensitive GH123 and TRIM5α-resistant GH123/Q and GH123/A [21]. GH123/Q and GH123/A encode CA (Gln120) and CA (Ala120), respectively [15]. Contrary to our expectation, the predicted L4/5 conformations of the NTDs of the NMC842 CA and GH123/G CA differed from those of TRIM5α-resistant GH123/Q and GH123/A, better resembling that predicted for the CA NTD encoded by TRIM5α-sensitive GH123 (Fig. 7). Indeed, the calculated probability of hydrogen bond formation between L4/5 and L6/7 was even lower for the CAs of GH123/G (20.80%) and NMC842 (30.58%) compared to that of GH123 (44.6%). These results suggest that Gly119 endows the CRF01_AB CA NTD with unique structural properties.

### Steric Locations of HIV-2 CRF01_AB-specific Amino Acid Substitutions

**Figure 8:**
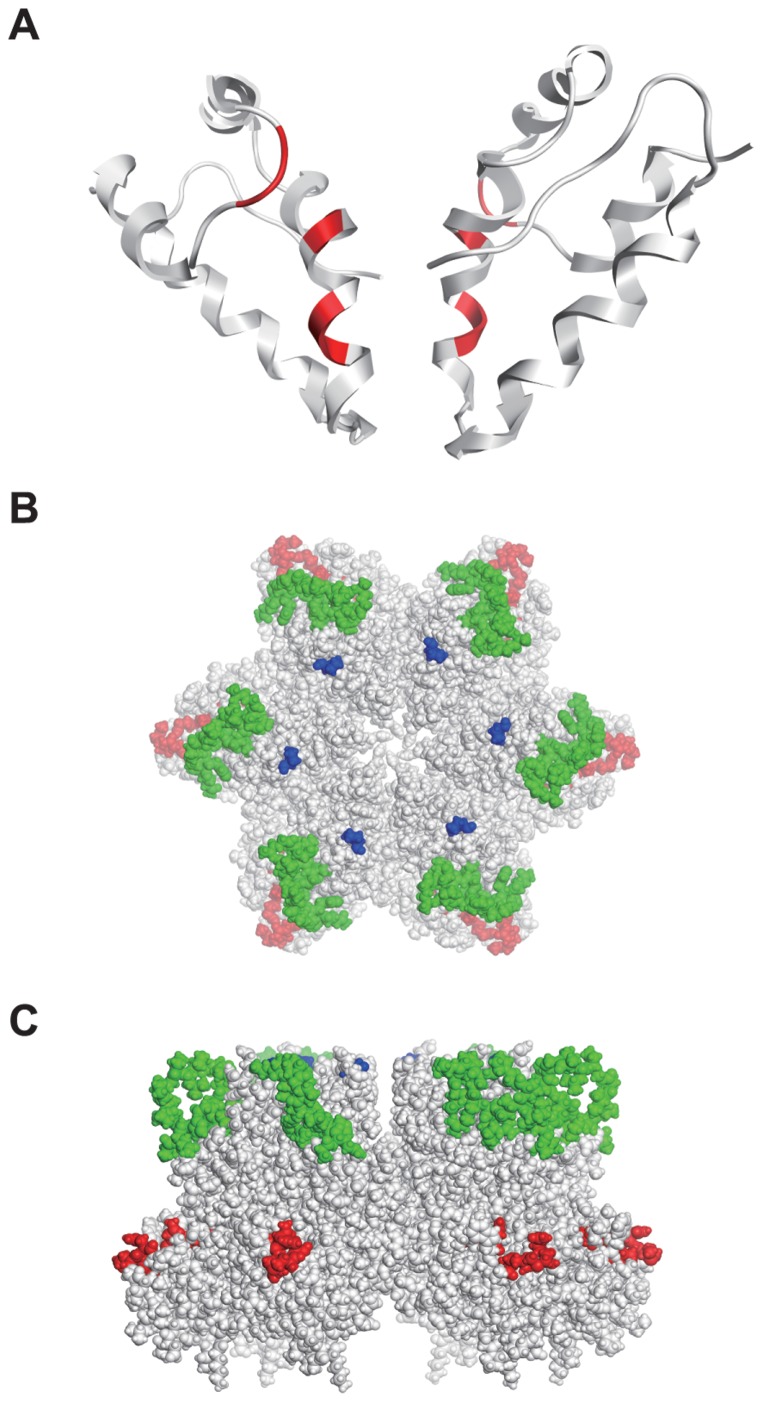
Structural models of the HIV-2 capsid C-terminal domain in dimeric form (A) and the HIV-2 GH123 capsid hexamer (B and C). (A) The C-terminal domain dimer model (from the amino acid position 150 to 219) of HIV-2 capsid (CA) is based on the viral sequence of NMC842. HIV-2 CRF01_AB-specific amino acid substitutions are shown in red. (B and C) The space-filling model of CA hexamer from the top (B) and side (C) is shown. Positions of HIV-2 CRF01_AB-specific amino acid substitutions are shown in red. L4/5 and 120P are shown in green and blue, respectively.

As noted above, HIV-2 CRF01_AB strains have several specific amino acid substitutions at the C-terminal domain (CTD) of CA (Fig. 1, shown in red); these substitutions were necessary for the potent resistance of these isolates against human TRIM5α (Fig. 6). Previously, we suggested that magnitudes of the computationally calculated binding energies of the CA CTD dimer models tend to be significantly greater in the TRIM5α-less-sensitive HIV-2s in West Africa [18]. To examine if the HIV-2 CRF01_AB-specific amino acid substitutions in CA CTD could influence the CTD-CTD dimer stability, we constructed the CA CTD dimer model of HIV-2 CRF01_AB NMC842 by homology modeling and analyzed steric locations of the specific substitutions and binding energies of the CTD dimer model. In the CA CTD dimer model of NMC842, HIV-2 CRF01_AB-specific amino acid substitutions are located in helix 9 and in the loop between helices 10 and 11, and all appeared to be situated near but distinct from the CTD-CTD dimer interface (Fig. 8A). The predicted binding energy of the CTD-CTD dimer model of the NMC842 isolate (79.6 kcal/mole) was similar to that reported in TRIM5α sensitive viruses [18]. The results may imply that the HIV-2 CRF01_AB-specific amino acid substitutions in CTD do not necessarily influence the CTD-CTD dimer stability of the TRIM5α sensitive virus.

To further obtain structural insights into the roles of these CRF01_AB-specific mutations, we analyzed their steric locations in the CA hexamer. In the hexamer model of GH123 CA that we previously constructed based on the HIV-1 CA hexamer [28], HIV-2 CRF01_AB-specific amino acid substitutions in CTD form clusters and are located at the outermost part of the hexamer (Fig. 8B and C). Notably, these substitutions exist directly under the L4/5 of neighboring CA (Fig. 8C), and most of them are clearly visible from right above (Fig. 8B). These results raise a possibility that HIV-2 CRF01_AB-specific amino acid substitutions in CA CTD may be exposed to and accessible from the outside of the viral core.

## Discussion

In the present study, we have shown that the CA of HIV-2 CRF01_AB isolates have a unique feature distinct from that of other HIV-2 strains; CRF01_AB-specific sequences conferred strong resistance to human TRIM5α. In addition to the previously identified role of amino acid 119 of the CA NTD, CRF01_AB-specific amino acid substitutions in the CA CTD also were necessary for strong resistance to human TRIM5α. These amino acid substitutions in CA CTD may be exposed to and accessible from the outside of the viral core.

Retroviral CA is known to form hexamers [29]. The CTD domain of retroviral capsid protein participates in CA dimerization, where intermolecular CTD-CTD interactions are mediated by symmetric, parallel dimerization of helix 9 from the CTD domains of adjacent hexamers [30]. This dimerization process is prerequisite for assemblies of multiple hexamers [29]. Previously, we found that the computationally calculated binding energies of the CA CTD dimer models could have positive relations with the TRIM5α susceptibilities of HIV-2s in West Africa [18]. We therefore calculated here the binding energy of the CTD-CTD dimer model of the NMC842 using computational method. However, the predicted binding energy of the CTD-CTD dimer of the NMC842 isolate was rather similar to that reported in TRIM5α sensitive viruses [18]. Therefore, previously undescribed mechanisms may be involved in the TRIM5α resistance of the HIV-2 CRF01_AB.

A possible mechanism for the findings may be that the CRF01_AB-specific substitutions influence directly or indirectly the structural properties of an interaction surface for the TRIM5α mediated inhibition. In this regard, we previously suggested with SIV that not only the NTD but also the CTD might constitute an intermolecular interaction surface [31]. Similarly, HIV-2 may have such interaction surface in CTD domain, and the surface may be used for the TRIM5α-mediated inhibition. Results on the steric locations of the CRF01_AB-specific substitutions in the hexamer model support this possibility (Fig. 7B and C). A preliminary modeling study of the assemblies of the CA hexamers also have supported this possibility: the NTDs are apart from each other among the hexamers, which allows to form accessible surface on the CTDs (data not shown), as suggested with Rous sarcoma virus CA [32]. Therefore, it would be interesting to examine whether HIV-2 CRF01_AB-specific amino acid substitutions in CTD could constitute a binding cleft for the TRIM5α itself or others involved in TRIM5α medicated inhibition in the assemblies of multiple CA hexamers in the viral core. Further study is necessary to address this issue.

Previously, we showed that the amino acid replacements at CA residue 119 affected the conformation of the neighboring L4/5, and that TRIM5α-sensitive viruses had a shared L4/5 conformation that was associated with a decreased probability of hydrogen bonding between L4/5 and L6/7 [21]. Although GH123/G and 842 showed resistance to TRIM5α, the calculated probability of hydrogen bond formation between L4/5 and L6/7 was lower than that calculated for the CAs of other TRIM5α-resistant viruses, including that from GH123/Q (55.15%) and GH123/A (64.47%) [21]. The conformations of L4/5 in the CAs of GH123/G and 842 also were similar to those of TRIM5α-sensitive viruses, and were distinct from those of the CAs of TRIM5α-resistant viruses. These characteristics of GH123/G and 842 were similar to those of GH123/E and GH123/D, mutant GH123 clones encoding glutamic acid and aspartic acid (respectively) at the residue corresponding to position 119 of HIV-2 CRF01_AB strains [21]. Although glutamic acid and aspartic acid have not been observed at this CA residue in HIV-2 isolated clinically, both GH123/E and GH123/D showed resistance against CM TRIM5α. In contrast to the CAs of GH123/Q and GH123/A, the CAs of both GH123/E and GH123/D show reduced likelihoods of hydrogen bond formation between the L4/5 and L6/7, and the L4/5 conformations were predicted to be similar to those of the CAs of TRIM5α-sensitive viruses. Therefore, our present results extend our previous observations, and additionally imply that the Gly119 of HIV-2 CRF01_AB CA prevents binding by TRIM5α, probably due to the small size of the glycine side chain. It is possible that the shared conformation of L4/5 might have some advantages in utilizing certain cellular factor(s) that bind CA. Our structural data suggests that HIV-2 CRF01_AB strains are highly adapted, since these strains have acquired potent resistance against TRIM5α without losing the shared L4/5 conformation.

In the case of GH123/E, disruption of the hydrogen bond between L4/5 and L6/7 by substitution of alanine for aspartic acid at position 97 (D97A) did not alter the resistant phenotype of GH123/E [21], while the same substitution almost completely abolished the replicative ability of GH123/G (data not shown). This result further demonstrates the unique status of GH123/G, since D97A substitution did not cause such a drastic reduction of replicative ability in GH123, GH123/Q, and GH123/A [21]. The basis for the difference between GH123/G and other variants is unclear; further mutational studies will be necessary to elucidate detailed interactions between L4/5 and L6/7, and to define the contribution of these sequences to viral replication and TRIM5α sensitivity.

In the Los Alamos databases, almost all SIV isolates encode glutamine at the position corresponding to residue 119 of the HIV-2 CRF01_AB CA. It is likely that the sequential mutation from glutamine (coded as CAA or CAG) to proline (CCA or CCG; underlines denote single nucleotide changes) and then to alanine (GCA, GCG) occurred after transmission of the monkey virus to the human population. The nature of the genetic code suggests that the Gly119-encoding virus (GGA or GGG codon) derived from the Ala119-encoding virus, implying that the viruses with glycine are highly adapted, as also discussed above. A single HIV-2 strain encoding glycine at the 119th CA residue was found in the Los Alamos databases; this strain (7312A) was isolated from a symptomatic 32-years-old man [33], and also was a recombinant between groups A and B (Fig. 1 and 2). This recombinant virus exhibits a genomic organization similar to that of NMC307, NMC716, and NMC842. At present, we do not know whether the emergence of glycine at the 119th position of CA is unique to HIV-2 CRF01_AB. It will be critical to assess the emergence of Gly119 viruses within HIV-2 groups A and B.

It is generally believed that HIV-2 is less pathogenic than HIV-1, and the number of HIV-2 cases is now gradually decreasing in West Africa. However, NMC307, NMC716, and NMC842 were recovered from patients at an advanced stage of AIDS with low CD4+ cell counts and high HIV-2 VLs [19]. It is possible that these HIV-2 CRF01_AB strains are highly pathogenic, unlike other HIV-2 strains. Careful epidemiological and virological studies are necessary to test this hypothesis. In the present study, we found that HIV-2 CRF01_AB CA confers strong resistance to human TRIM5α. In the Caio HIV-2 cohort in West Africa, non-proline residues at position 119 were significantly associated with elevated plasma HIV-2 load [18]. Therefore, resistance to TRIM5α may at least partially explain why these 3 patients in Japan developed AIDS so rapidly, although the possible effects of mutations in regions (e.g., *env*, *vif*, *nef* and the long terminal repeats) other than those that encode CA cannot be fully excluded at present. Our results also suggest that resistance to TRIM5α might be a new marker for the pathogenic potential of HIV-2. The possible emergence of a highly pathogenic HIV-2 strain is an ongoing concern, given that retroviruses can easily evolve to evade host defenses.

## Materials and Methods

### Phylogenetic Tree Analysis

Multiple sequence alignment was performed using the software CLUSTALW version 2.1. Phylogenetic trees were constructed using the neighbor-joining method. Bootstrap probabilities were calculated by 1000 iterations [34].

### Cell Culture

The human 293T [35] and feline CRFK [36] cells were maintained in Dulbecco’s Modified Eagle medium. The human T-cell line CEM-SS [37] was maintained in RPMI medium. All media were supplemented with 10% fetal bovine serum and 1% penicillin-streptomycin.

### Plasmid Construction

Recombinant HIV-2 GH123 clones containing the entire CA sequence of the isolates NMC716 or NMC842 (716 or 842, respectively) and 716 or 842 with proline substitutions at the 178th position (716GPP or 842GPP, respectively) were generated by PCR-based mutagenesis. The GH123/G virus was described previously [21]. The 0.6-kb *Hind*III-*Xho*I fragment of 842 was replaced with the corresponding fragment of GH123/G, and the resulting plasmid was designated 842Hind. Infectious viruses were prepared by transfection of 293T cells with the resulting proviral DNA clones. Viral titers were determined by measuring P25 (CA) with a RetroTek antigen ELISA kit (ZeptoMetrix, Buffalo, NY).

To construct the wild-type and mutant HIV-2 clones encoding GFP, the 1.6-kb *Kpn*I-*Xho*I fragment (which encodes the MA, CA and p6) of GH123, 842, or GH123/G, was transferred to pROD-env(-)-GFP [25], a clone in which the *env* gene is disrupted, and the GFP gene was inserted into the *nef* region. Infectious viruses were prepared by transfection of 293T cells with proviral DNA clones together with the pMD2G plasmid encoding VSV-G. Viral titers were determined as above.

Construction of recombinant SeV encoding C-terminally HA-tagged CM TRIM5α (CM-TRIM5α-SeV), Rh TRIM5α (Rh-TRIM5α-SeV), human TRIM5α (Hu-TRIM5α-SeV), and CM TRIM5α lacking the PRYSPRY domain (CM-SPRY(-)-SeV) were described previously [10], [15], [22].

### Viral Infection

CEM-SS cells (1×10^6^) were infected with SeVs encoding the respective TRIM5α proteins at a multiplicity of infection of 10 plaque-forming units per cell and incubated at 37°C for 9 h. Aliquots of 1×10^5^ cells were then superinfected with GH123, GH123/G, 716, 716GPP, 842, 842GPP, or 842Hind virus. Each superinfection used a titer of virus corresponding to 20 ng of p25 (CA). Experiment was performed three separate times with duplicate samples. For viral infection of cells producing physiological levels of TRIM5α, TRIM5α knock-down Jurkat cells (TRIM5α-KD Jurkat) and control cells (Luci-siRNA Jurkat) were infected with GH123, GH123/G, 716, or 842 virus. Each infection used a titer of virus corresponding to 100 ng of p25. The culture supernatants were collected periodically, and the level of p25 (CA) was measured as described above. Experiment was performed two separate times with duplicate samples.

### Western Blot

CEM-SS cells (1×10^6^) infected with recombinant SeVs expressing HA-tagged TRIM5α proteins were lysed in lysis buffer (50 mM Tris-HCl, pH 7.5, 150 mM NaCl, 1% Nonidet P40, 0.5% sodium deoxycholate). TRIM5α proteins in the lysates were subjected to sodium dodecyl sulfate-polyacrylamide gel electrophoresis. Proteins in the gel were then electronically transferred onto a membrane (Immobilon; Millipore, Billerica, MA). Blots were blocked and probed with anti-HA high-affinity rat monoclonal antibody (Roche, Indianapolis, IN) overnight at 4°C. Blots were then incubated with peroxidase-conjugated anti-rat IgG (American Qualex, San Clemente, CA), and bound antibodies were visualized with a Chemilumi-One chemiluminescent kit (Nacalai Tesque, Kyoto, Japan).

### Single-round Infection Assay

SeV-infected CRFK cells (4×10^4^) were infected with a titer of pROD-env(-)-GFP derivative virus corresponding to 500 ng of p25 (CA). Two days after infection, the cells were fixed by formaldehyde, and GFP-producing cells were counted by flow cytometry. Experiment was performed three separate times with triplicate samples.

### Molecular Modeling and MD Simulation

We used molecular dynamic (MD) simulations [38] to analyze the structural dynamics of the HIV-2 CA NTDs. First, initial CA structures for MD simulation were constructed by homology modeling [39] using the Molecular Operating Environment, MOE (Chemical Computing Group Inc., Montreal, Canada) as described previously [15], [40]. We used the high-resolution crystal structure of the HIV-2 CA NTD at a resolution of 1.25Å (PDB code: 2WLV) [16] as the modeling template. Structural dynamics of these HIV-2 CA models in an aqueous environment were analyzed using MD simulations with the SANDER module in the AMBER 9 program package [41] and the AMBER99SB force field with the TIP3P water model [42]. Bond lengths involving hydrogen were constrained with SHAKE [43] and the time step for all MD simulations was set to 2 fs. After heating calculations for 20 ps to 310 K using the NVT ensemble, the simulations were executed using the NPT ensemble at 1 atm and 310 K for 20 ns. Hydration analyses were performed using the ptraj module in AMBER. A maximum cut-off angle of 120.0° and cut-off length of 3.5 Å were used in hydrogen bond definitions.

For the CTD dimer model of HIV-2 CRF01_AB NMC842, a crystal structure of the HIV-1 CA protein was used as the template for the modeling; the dimer of CA C-terminal domain at a resolution of 1.70 Å (PDB code: 1A8O) [17]. The amino acid sequence identity of HIV-1 (1A8O) and HIV-2 CA (NMC842 in this study) is about 76%. The sequence similarity is sufficient to construct a structural model with an r.m.s. deviation of approximately 1.5 Å for the main chain between the predicted and actual structures [39]. The 3-D structures were optimized thermodynamically by energy minimization using MOE and an AMBER99 force field [44] and further refined the physically unacceptable local structures on the basis of evaluation of unusual dihedral angles, *phi* and *psi*, by the Ramachandran plot using MOE. The binding energies of the CA dimer models, E_bind_, were calculated as described elsewhere [45], [46], using the formula E_bind_ = E_dimer_–2E_monomer_, where E_dimer_ is the energy of the CA dimer; E_monomer_ is the energy of the CA monomer.

### Conclusions

The CA of HIV-2 CRF01_AB isolates have a unique feature distinct from that of other HIV-2 strains; CRF01_AB-specific sequences conferred strong resistance to human TRIM5α. CRF01_AB-specific amino acid substitutions in the CA CTD were necessary for strong resistance to human TRIM5α.

## Supporting Information

Figure S1 **Western blot analysis of TRIM5α proteins.** HA-tagged TRIM5α proteins in lysate of CEM-SS cells infected with recombinant SeV were visualized by western blotting with an antibody against HA. S(-), Hu, CM, and Rh denote CM SPRY(-), human, cynomolgus monkey, and rhesus TRIM5α, respectively. Molecular weight makers are shown on the left.
(EPS)

## References

[1] Rowland-Jones SL, Whittle HC (2007) Out of Africa: what can we learn from HIV-2 about protective immunity to HIV-1? Nat Immunol 8: 329–331. 17375091 10.1038/ni0407-329

[2] Gao F, Bailes E, Robertson DL, Chen Y, Rodenburg CM, et al (1999) Origin of HIV-1 in the chimpanzee Pan troglodytes troglodytes. Nature 397: 436–441. 9989410 10.1038/17130

[3] Huet T, Cheynier R, Meyerhans A, Roelants G, Wain-Hobson S (1990) Genetic organization of a chimpanzee lentivirus related to HIV-1. Nature 345: 356–359. 2188136 10.1038/345356a0

[4] Gottlieb GS, Sow PS, Hawes SE, Ndoye I, Redman M, et al (2002) Equal plasma viral loads predict a similar rate of CD4+ T cell decline in human immunodeficiency virus (HIV) type 1- and HIV-2-infected individuals from Senegal, West Africa. J Infect Dis 185: 905–914. 11920314 10.1086/339295

[5] Damond F, Worobey M, Campa P, Farfara I, Colin G, et al (2004) Identification of a highly divergent HIV type 2 and proposal for a change in HIV type 2 classification. AIDS Res Hum Retroviruses 20: 666–672. 15242544 10.1089/0889222041217392

[6] Stremlau M, Owens CM, Perron MJ, Kiessling M, Autissier P, et al (2004) The cytoplasmic body component TRIM5alpha restricts HIV-1 infection in Old World monkeys. Nature 427: 848–853. 14985764 10.1038/nature02343

[7] Sebastian S, Luban J (2005) TRIM5alpha selectively binds a restriction-sensitive retroviral capsid. Retrovirology 2: 40. 15967037 10.1186/1742-4690-2-40 PMC1166576

[8] Stremlau M, Perron M, Lee M, Li Y, Song B, et al (2006) Specific recognition and accelerated uncoating of retroviral capsids by the TRIM5alpha restriction factor. Proceedings of the National Academy of Sciences of the United States of America 103: 5514–5519. 16540544 10.1073/pnas.0509996103 PMC1459386

[9] Reymond A, Meroni G, Fantozzi A, Merla G, Cairo S, et al (2001) The tripartite motif family identifies cell compartments. Embo J 20: 2140–2151. 11331580 10.1093/emboj/20.9.2140 PMC125245

[10] Nakayama EE, Miyoshi H, Nagai Y, Shioda T (2005) A specific region of 37 amino acid residues in the SPRY (B30.2) domain of African green monkey TRIM5alpha determines species-specific restriction of simian immunodeficiency virus SIVmac infection. Journal of Virology 79: 8870–8877. 15994780 10.1128/JVI.79.14.8870-8877.2005 PMC1168783

[11] Hatziioannou T, Perez-Caballero D, Yang A, Cowan S, Bieniasz PD (2004) Retrovirus resistance factors Ref1 and Lv1 are species-specific variants of TRIM5alpha. Proceedings of the National Academy of Sciences of the United States of America 101: 10774–10779. 15249685 10.1073/pnas.0402361101 PMC490010

[12] Keckesova Z, Ylinen LM, Towers GJ (2004) The human and African green monkey TRIM5alpha genes encode Ref1 and Lv1 retroviral restriction factor activities. Proceedings of the National Academy of Sciences of the United States of America 101: 10780–10785. 15249687 10.1073/pnas.0402474101 PMC490011

[13] Perron MJ, Stremlau M, Song B, Ulm W, Mulligan RC, et al (2004) TRIM5alpha mediates the postentry block to N-tropic murine leukemia viruses in human cells. Proceedings of the National Academy of Sciences of the United States of America 101: 11827–11832. 15280539 10.1073/pnas.0403364101 PMC511059

[14] Nakayama EE, Shioda T (2010) Anti-retroviral activity of TRIM5 alpha. Rev Med Virol 20: 77–92. 20049904 10.1002/rmv.637

[15] Song H, Nakayama EE, Yokoyama M, Sato H, Levy JA, et al (2007) A single amino acid of the human immunodeficiency virus type 2 capsid affects its replication in the presence of cynomolgus monkey and human TRIM5alphas. Journal of Virology 81: 7280–7285. 17475650 10.1128/JVI.00406-07 PMC1933286

[16] Price AJ, Marzetta F, Lammers M, Ylinen LM, Schaller T, et al (2009) Active site remodeling switches HIV specificity of antiretroviral TRIMCyp. Nat Struct Mol Biol 16: 1036–1042. 19767750 10.1038/nsmb.1667 PMC3556581

[17] Gamble TR, Yoo S, Vajdos FF, von Schwedler UK, Worthylake DK, et al (1997) Structure of the carboxyl-terminal dimerization domain of the HIV-1 capsid protein. Science 278: 849–853. 9346481 10.1126/science.278.5339.849

[18] Onyango CO, Leligdowicz A, Yokoyama M, Sato H, Song H, et al (2010) HIV-2 capsids distinguish high and low virus load patients in a West African community cohort. Vaccine 28S2: B60–B67. 10.1016/j.vaccine.2009.08.060 20510746

[19] Ibe S, Yokomaku Y, Shiino T, Tanaka R, Hattori J, et al (2010) HIV-2 CRF01_AB: first circulating recombinant form of HIV-2. J Acquir Immune Defic Syndr 54: 241–247. 20502347 10.1097/QAI.0b013e3181dc98c1

[20] Marlink R, Kanki P, Thior I, Travers K, Eisen G, et al (1994) Reduced rate of disease development after HIV-2 infection as compared to HIV-1. Science 265: 1587–1590. 7915856 10.1126/science.7915856

[21] Miyamoto T, Yokoyama M, Kono K, Shioda T, Sato H, et al (2011) A single amino acid of human immunodeficiency virus type 2 capsid protein affects conformation of two external loops and viral sensitivity to TRIM5alpha. PloS one 6: e22779. 21829511 10.1371/journal.pone.0022779 PMC3145752

[22] Kono K, Song H, Shingai Y, Shioda T, Nakayama EE (2008) Comparison of anti-viral activity of rhesus monkey and cynomolgus monkey TRIM5alphas against human immunodeficiency virus type 2 infection. Virology 373: 447–456. 18201746 10.1016/j.virol.2007.12.022

[23] Wu X, Anderson JL, Campbell EM, Joseph AM, Hope TJ (2006) Proteasome inhibitors uncouple rhesus TRIM5alpha restriction of HIV-1 reverse transcription and infection. Proceedings of the National Academy of Sciences of the United States of America 103: 7465–7470. 16648264 10.1073/pnas.0510483103 PMC1464362

[24] Anderson JL, Campbell EM, Wu X, Vandegraaff N, Engelman A, et al (2006) Proteasome inhibition reveals that a functional preintegration complex intermediate can be generated during restriction by diverse TRIM5 proteins. Journal of Virology 80: 9754–9760. 16973579 10.1128/JVI.01052-06 PMC1617233

[25] Pertel T, Reinhard C, Luban J (2011) Vpx rescues HIV-1 transduction of dendritic cells from the antiviral state established by type 1 interferon. Retrovirology 8: 49. 21696578 10.1186/1742-4690-8-49 PMC3130655

[26] Sokolskaja E, Berthoux L, Luban J (2006) Cyclophilin A and TRIM5alpha independently regulate human immunodeficiency virus type 1 infectivity in human cells. Journal of Virology 80: 2855–2862. 16501094 10.1128/JVI.80.6.2855-2862.2006 PMC1395419

[27] Pertel T, Hausmann S, Morger D, Zuger S, Guerra J, et al (2011) TRIM5 is an innate immune sensor for the retrovirus capsid lattice. Nature 472: 361–365. 21512573 10.1038/nature09976 PMC3081621

[28] Kono K, Song H, Yokoyama M, Sato H, Shioda T, et al (2010) Multiple sites in the N-terminal half of simian immunodeficiency virus capsid protein contribute to evasion from rhesus monkey TRIM5alpha-mediated restriction. Retrovirology 7: 72. 20825647 10.1186/1742-4690-7-72 PMC2944288

[29] Pornillos O, Ganser-Pornillos BK, Kelly BN, Hua Y, Whitby FG, et al (2009) X-ray structures of the hexameric building block of the HIV capsid. Cell 137: 1282–1292. 19523676 10.1016/j.cell.2009.04.063 PMC2840706

[30] Ganser-Pornillos BK, Yeager M, Sundquist WI (2008) The structural biology of HIV assembly. Curr Opin Struct Biol 18: 203–217. 18406133 10.1016/j.sbi.2008.02.001 PMC2819415

[31] Inagaki N, Takeuchi H, Yokoyama M, Sato H, Ryo A, et al (2010) A structural constraint for functional interaction between N-terminal and C-terminal domains in simian immunodeficiency virus capsid proteins. Retrovirology 7: 90. 20955553 10.1186/1742-4690-7-90 PMC2964592

[32] Bailey GD, Hyun JK, Mitra AK, Kingston RL (2012) A structural model for the generation of continuous curvature on the surface of a retroviral capsid. J Mol Biol 417: 212–223. 22306463 10.1016/j.jmb.2012.01.014

[33] Gao F, Yue L, White AT, Pappas PG, Barchue J, et al (1992) Human infection by genetically diverse SIVSM-related HIV-2 in west Africa. Nature 358: 495–499. 1641038 10.1038/358495a0

[34] Hillis DM, Bull JJ (1993) An empirical test of bootstrapping as a method for assessing confidence in phylogenetic analysis. Syst Biol 42: 182–192.

[35] Pear WS, Nolan GP, Scott ML, Baltimore D (1993) Production of high-titer helper-free retroviruses by transient transfection. Proc Natl Acad Sci U S A 90: 8392–8396. 7690960 10.1073/pnas.90.18.8392 PMC47362

[36] Lasfargues EY, Lasfargues JC, Dion AS, Greene AE, Moore DH (1976) Experimental infection of a cat kidney cell line with the mouse mammary tumor virus. Cancer Res 36: 67–72. 55305

[37] Sheehy AM, Gaddis NC, Choi JD, Malim MH (2002) Isolation of a human gene that inhibits HIV-1 infection and is suppressed by the viral Vif protein. Nature 418: 646–650. 12167863 10.1038/nature00939

[38] Dodson GG, Lane DP, Verma CS (2008) Molecular simulations of protein dynamics: new windows on mechanisms in biology. EMBO Rep 9: 144–150. 18246106 10.1038/sj.embor.7401160 PMC2246404

[39] Baker D, Sali A (2001) Protein structure prediction and structural genomics. Science 294: 93–96. 11588250 10.1126/science.1065659

[40] Shirakawa K, Takaori-Kondo A, Yokoyama M, Izumi T, Matsui M, et al (2008) Phosphorylation of APOBEC3G by protein kinase A regulates its interaction with HIV-1 Vif. Nat Struct Mol Biol 15: 1184–1191. 18836454 10.1038/nsmb.1497

[41] Case DA, Darden TA, Cheatham I, T.E., Simmerling CL, Wang JR, et al.. (2006) AMBER 9. University of California, San Francisco.

[42] Hornak V, Abel R, Okur A, Strockbine B, Roitberg A, et al (2006) Comparison of multiple Amber force fields and development of improved protein backbone parameters. Proteins 65: 712–725. 16981200 10.1002/prot.21123 PMC4805110

[43] Ryckaert J-P, Ciccotti G, Berendsen HJC (1977) Numerical integration of the cartesian equations of motion of a system with constraints: Molecular dynamics of n-alkanes. J Comput Phys 23: 327–341.

[44] Ponder JW, Case DA (2003) Force fields for protein simulations. Adv Protein Chem 66: 27–85. 14631816 10.1016/s0065-3233(03)66002-x

[45] Lee K, Chu CK (2001) Molecular modeling approach to understanding the mode of action of L-nucleosides as antiviral agents. Antimicrob Agents Chemother 45: 138–144. 11120956 10.1128/AAC.45.1.138-144.2001 PMC90251

[46] Kinomoto M, Yokoyama M, Sato H, Kojima A, Kurata T, et al (2005) Amino acid 36 in the human immunodeficiency virus type 1 gp41 ectodomain controls fusogenic activity: implications for the molecular mechanism of viral escape from a fusion inhibitor. Journal of Virology 79: 5996–6004. 15857986 10.1128/JVI.79.10.5996-6004.2005 PMC1091722

